# A standardized protocol for manually segmenting stroke lesions on high-resolution T1-weighted MR images

**DOI:** 10.3389/fnimg.2022.1098604

**Published:** 2023-01-10

**Authors:** Bethany P. Tavenner, Miranda R. Donnelly, Giuseppe Barisano, Sook-Lei Liew

**Affiliations:** ^1^Chan Division of Occupational Science and Occupational Therapy, University of Southern California, Los Angeles, CA, United States; ^2^Department of Neurosurgery, Stanford University, Stanford, CA, United States; ^3^Keck School of Medicine, Mark and Mary Stevens Neuroimaging and Informatics Institute, University of Southern California, Los Angeles, CA, United States

**Keywords:** stroke, manual segmentation, MRI segmentation, stroke lesion, segmentation protocol, T1w MRI, ITK-SNAP

## Abstract

Although automated methods for stroke lesion segmentation exist, many researchers still rely on manual segmentation as the gold standard. Our detailed, standardized protocol for stroke lesion tracing on high-resolution 3D T1-weighted (T1w) magnetic resonance imaging (MRI) has been used to trace over 1,300 stroke MRI. In the current study, we describe the protocol, including a step-by-step method utilized for training multiple individuals to trace lesions (“tracers”) in a consistent manner and suggestions for distinguishing between lesioned and non-lesioned areas in stroke brains. Inter-rater and intra-rater reliability were calculated across six tracers trained using our protocol, resulting in an average intraclass correlation of 0.98 and 0.99, respectively, as well as a Dice similarity coefficient of 0.727 and 0.839, respectively. This protocol provides a standardized guideline for researchers performing manual lesion segmentation in stroke T1-weighted MRI, with detailed methods to promote reproducibility in stroke research.

## Introduction

Segmenting stroke lesions is a common procedure in stroke neuroimaging research. Lesion masks, whether created with automated processes or manual tracing, provide information on lesion location, size, and overlap with regions of interest. These measures are consistently utilized as biomarkers for rehabilitation outcomes (Feng et al., 2015; Boyd et al., 2017; Kim and Winstein, 2017), and provide critical information for characterizing stroke damage.

Manual methods for lesion segmentation continue to be the gold standard for many stroke researchers. While some automated methods exist, they require significant manual correction, and many do not yet provide the accuracy desired for research applications (Ito et al., 2019; Liew et al., 2022a). A key challenge of stroke lesion segmentation is the high variability in size and shape, particularly for small lesions, and distinguishing stroke lesions from other abnormalities, such as white matter hyperintensities or perivascular spaces. These challenges create difficulty for both automated and manual segmentation processes.

Manual tracing is a time-consuming and subjective process, resulting in intra-rater and inter-rater variability. In addition, publications often lack detailed methods on how lesions in a dataset were traced, and there is currently no formally published protocol for stroke T1-weighted MRI lesion tracing. Although we have previously shared guidelines for lesion segmentation informally (Liew et al., 2018, 2022a), here we aim to provide a standardized protocol to decrease the subjectivity of manual lesion tracing and increase the reliability of segmentations across researchers (especially in multi-site analyses).

Here, we share our protocol with detailed methods for manual stroke lesion segmentation, including training multiple tracers on our pipeline for processing stroke lesions in high-resolution T1w MRIs. The protocol includes resources for those with limited stroke neuroanatomy knowledge and a detailed process used for testing reliability between tracers to ensure consistency and data quality. This protocol was used in the development of ATLAS v2.0, a multi-site dataset of 1,271 stroke T1w MRIs and manually-segmented lesion masks (Liew et al., 2022a), and has been used to generate many segmentations since then. In this paper, we also examine the intra-rater and inter-rater reliability of multiple tracers using this protocol.

## Methods and analysis

### Image acquisition

All T1w MRI images that were used in the development of this protocol are from the ENIGMA Stroke Recovery working group (Liew et al., 2022b). Scanner type and sample header information (acquisition parameters) can be found in the supplementary information. Images used in the training were specifically chosen to represent lesions of various sizes and locations, creating a diverse set of training examples and reflecting the high variability of stroke lesion pathology. Similarly, control subjects included in the training were chosen to demonstrate a range of visible pathology from non-stroke subjects. T1w images were used over other modalities because this is a primary modality collected and used to delineate lesion damage in stroke rehabilitation research (Liew et al., 2022a). It also aligns with the goals of the ENIGMA Stroke Recovery Working Group to harmonize large, multi-site T1w MRI data for meta- and mega-analyses (Liew et al., 2018, 2022b).

### Protocol overview

**Figure 1:**
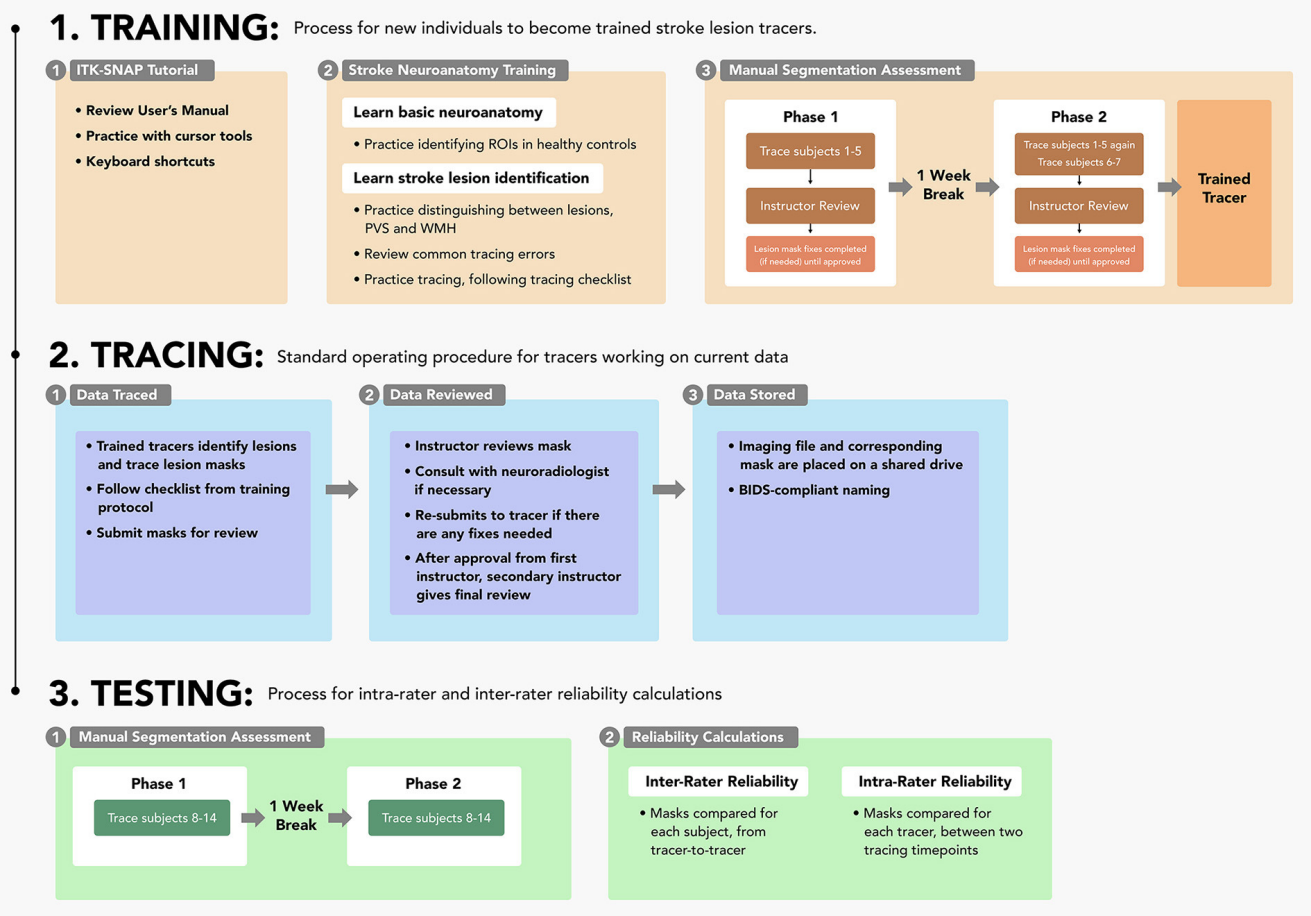
Protocol sections: A flowchart outlining each of the three parts of the protocol (training, tracing, and testing) and their respective steps.

This protocol is composed of three main sections: (1) the training process for individuals to learn to trace lesions, (2) the tracing pipeline for trained tracers, and (3) the testing steps for measuring intra-rater and inter-rater reliability. Details surrounding the entire protocol timeline are detailed in Figure 1. This protocol is shaped by our experience over the last 10 years of manually tracing stroke lesions; frequent questions from trainees have informed content in the basic neuroanatomy guide. It is important to note that stroke lesion tracing is a subjective process due to variations in stroke lesions and lesion border identification; this protocol is based on our experience with T1w MRI data in the ENIGMA Stroke Recovery Working Group and may not be generalizable to the entire stroke population.

### Training

Prior to tracing lesions on new data, all trainees were required to complete a training protocol, which includes (1) instructions on using the ITK-SNAP segmentation software, (2) lessons in stroke neuroanatomy and lesion identification, and (3) a manual segmentation assessment. Details and training resources for this protocol are available on GitHub (https://github.com/npnl/ATLAS/tree/master/Lesion%20Tracing%20Training).

#### Step 1: ITK-SNAP tutorial

**Figure 2:**
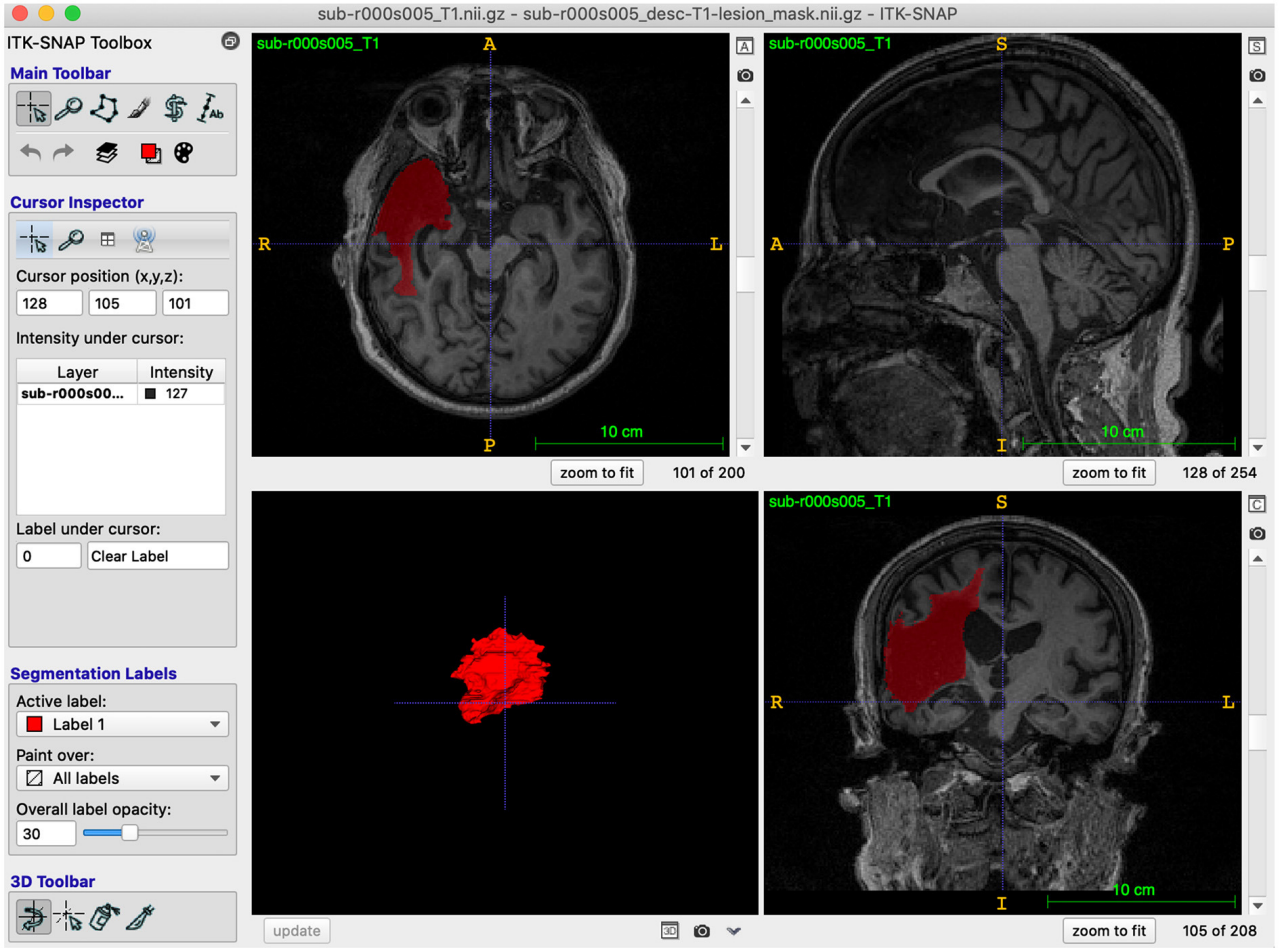
ITK-SNAP software: A screenshot example of the ITK-SNAP interface, demonstrating all three anatomical views and a 3D rendering of the traced segmentation.

Lesions were traced on ITK-SNAP (Yushkevich et al., 2006; Yushkevich and Gerig, 2017; version 3.8.0), which has an interface that is helpful for those new to neuroanatomy and a semi-automated lesion interpolation tool used for larger lesions (Figure 2). The interface has multiple configuration options, enabling the selection of viewing one anatomical view (coronal, axial, sagittal) at a time or all three on the same screen, and a 3D rendering image of the segmentation. Trainees completed tutorial steps from the ITK-SNAP user's manual (http://www.itksnap.org/docs/fullmanual.php) to familiarize themselves with tracing tools and practice tracing using different cursors and the interpolation tool. Trainees were encouraged to learn ITK-SNAP keyboard shortcuts (http://www.itksnap.org/pmwiki/pmwiki.php?n=Documentation.KeyboardShortcuts) to increase efficiency in the tracing process.

#### Step 2: Stroke neuroanatomy training

**Figure 3:**
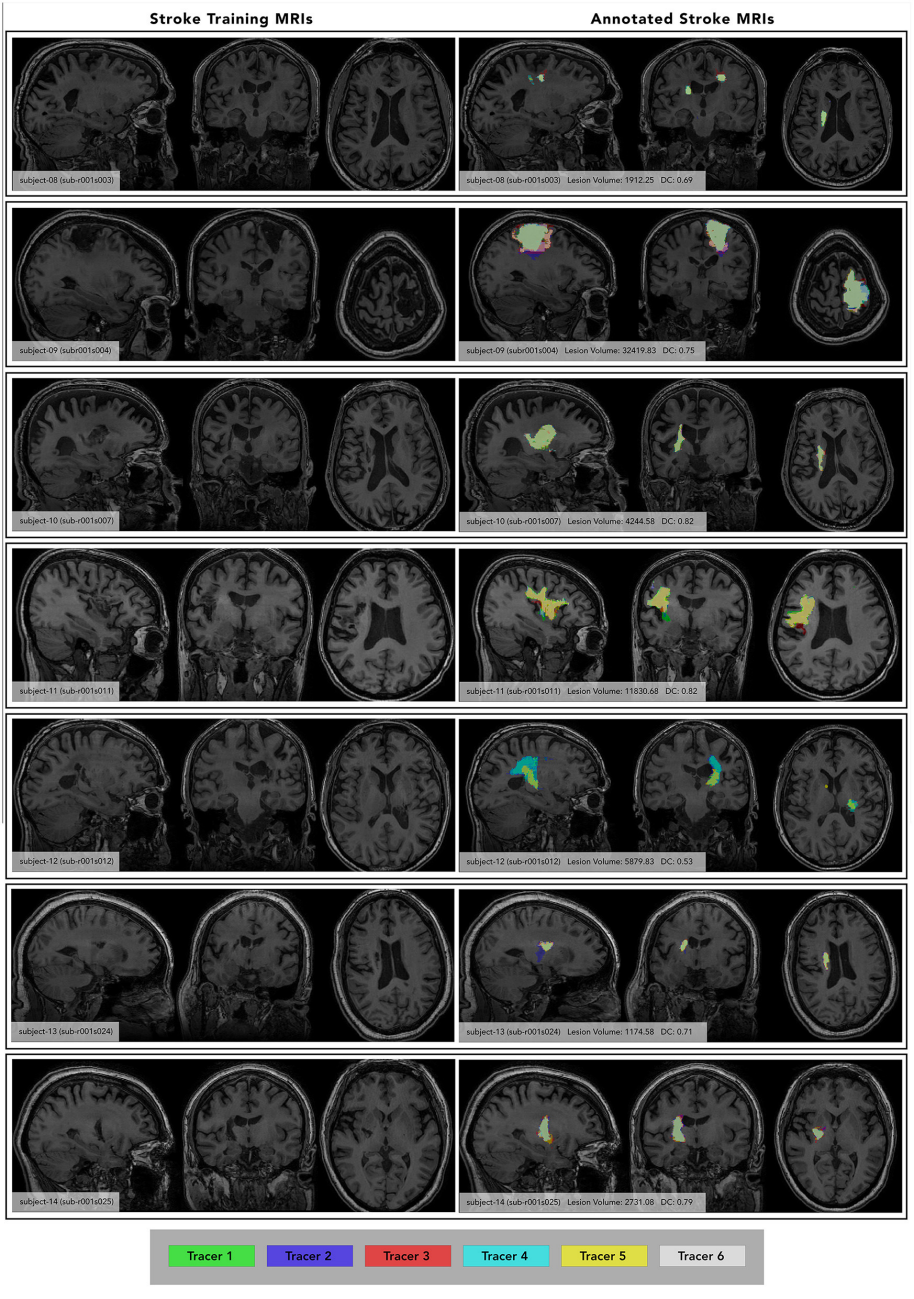
Individual Subject Stroke Lesions with and without lesion masks. **(Left)** images of each subject at maximum lesion width, with subject number and ATLAS subject ID included for reference. **(Right)** annotated images of each subject at the same maximum lesion width, showing segmentations from each tracer (color-coded key at the bottom). Subject number, average lesion volume (averaged between both phases) and inter-rater DC listed. Subject ID from the ATLAS dataset is also included for reference. Only segmentations from Phase 1 are shown, each tracer's segmentation is overlaid in a different color.

Next, trainees were taught neuroanatomical structures of healthy brains and general steps for stroke lesion identification. Trainees were provided T1w MRIs of 5 different non-stroke healthy control subjects and viewed them in ITK-SNAP to gain a basic understanding of what a T1w brain MRI looks like. Trainees viewed instructional materials, including videos of example tracings and a presentation about neuroanatomy that highlighted regions of interest for stroke. In our experience, it can be challenging for tracers to distinguish the borders between stroke lesions and cerebrospinal fluid in regions including the ventricles or insula, likely because of the similar signal intensity. An example of this can be seen in Figure 3 (subject 11), in which the boundary between the insula and lesion was variable between tracers. In addition, regions of interest such as the basal ganglia and sylvian fissure can be important for determining if pathology is a stroke lesion, as non-stroke pathology, such as enlarged perivascular spaces (PVS) and white matter hyperintensities (WMH), are more common in these areas (Bokura et al., 1998; Wardlaw et al., 2013). The training materials therefore focus on understanding normal anatomy in these regions. Trainees then familiarized themselves with finding these regions in the control subject images in all three anatomical views. Trainees had access to both a trained team member (henceforth “instructor”) (B.L. and M.D.) and a physician-scientist experienced in neuroradiology expert neuroradiologist (G.B.) for help with any issues that arose during this process.

To learn how to identify stroke lesions, trainees practiced distinguishing between stroke lesions, PVS and WMH, reviewed common tracing errors, and practiced tracing lesions while following a tracing checklist (https://github.com/npnl/ATLAS/blob/master/Lesion%20Tracing%20Training/Getting%20Started%20with%20Lesion%20Tracing_2022.pdf). In our experience, discerning between PVS, WMH and stroke lesions is a primary hurdle for tracers. Stroke is associated with enlarged PVS (Lau et al., 2017), causing difficulty in differentiation between PVS and stroke lesions. Information regarding the location, size, shape, and symmetry of the questioned area are critical in determining what kind of pathology is present (Wardlaw et al., 2013). To address this frequent challenge, neuroanatomy training lessons included a special focus on distinguishing between WMH, PVS and stroke lesion. All our instructional materials are available on GitHub (https://github.com/npnl/ATLAS/tree/master/Lesion%20Tracing%20Training). These training materials were developed in consultation with a physician-scientist experienced in neuroradiology (G.B.).

Finally, trainees were given a document that outlined several common tracing errors and a checklist for completing tracing in ITK-SNAP. Prior trainees with no prior experience with neuroimaging expressed that they often became visually overwhelmed by examining three different planes at the same time and often missed identifying lesions because of this. To address this, we included reminders in the document to encourage use of one plane at a time during lesion identification, toggling between views. We typically recommend using the axial view, followed by the coronal view, and finally the sagittal view, as the first two views allow for comparison between the lesioned and non-lesioned hemispheres. However, trainees were reminded that visual asymmetries in a scan can also be the result of physical head tilt in the scanner, thus using all three planes is recommended when making final decisions about the lesioned territory. In addition, lesion border inconsistencies were addressed by educating tracers to observe borders of other structures that might be easier to identify, such as the insula, ventricles, or subarachnoid space (a strategy commonly used in this situation was to go back and “erase” traced area that could be confidently accepted as *not* lesioned).

Trainees used the tracing checklist as a guide during the manual segmentation assessment, and afterward when tracing data. It includes steps that outline how to open and save segmentation files in ITK-SNAP and reminders of the thought process to follow when identifying and tracing a lesion. After locating the lesion to be traced, we recommend starting in a middle slice around the middle of the lesion using the cursor tool “smooth curve”, then tracing each slice (or every couple of slices for larger lesions, when using the “interpolate” tool) toward each end of the lesioned area. We also recommended frequently scrolling back and forth around the slice they are tracing on to ensure border consistency.

#### Step 3: Manual segmentation assessment

Once trainees completed Steps 1 and 2 and expressed confidence in identifying lesions and using the ITK-SNAP software, they began the manual segmentation assessment. The assessment consisted of two phases separated by 1 week. In Phase 1, trainees completed segmentations of subjects 1 through 5 and then received instructor feedback on their performance. Any adjustments or fixes were completed until satisfactory results were reached. Instructors referenced “answer key” segmentations (drawn by a separate trained team member and checked by an expert neuroradiologist) when determining if a trainee had satisfactory results. In Phase 2, 1 week later, trainees traced subjects 1 through 5 again (to measure intra-rater reliability) and 2 additional subjects for further practice in lesion identification. Trainees were instructed to not reference their Phase 1 segmentations when re-tracing. Instructor feedback was given, and adjustments completed as necessary. Segmentations were binarized, NIFTI files created on T1w, isometric NIFTI images. All lesions identified were included in a single lesion mask file and followed naming compliant to the Brain Imaging Data Structure (BIDS) convention (http://bids.neuroimaging.io/). After successfully tracing all seven subject segmentations, the trainee was considered a fully trained tracer.

### Tracing

The standard operating procedure for trained tracers working to segment lesions is similar to the training assessment. Imaging data is traced, reviewed by two separate instructors, and stored in BIDS-compliant naming and organization. Tracers identify and trace lesions, following the checklist given in training. They submit completed masks to be reviewed and receive feedback on their masks, completing any fixes if necessary. The first instructor approves masks, which are then cross-checked by a second instructor, who ensures the quality of the mask and records metadata about lesion size and location. To keep data organized, the lesion tracing team follows a detailed documentation process that guides communication and ensures the segmentation tracing process is streamlined and efficient. Data cohorts are organized on a shared drive, and individual tracers work on one cohort at a time. All tracers follow the tracing steps outlined in the training checklist, increasing consistency between tracers. A Slack channel is used for questions or clarifications, encouraging collaboration and learning for all team members.

### Testing

To test the quality of this protocol for the current paper, 6 trained tracers completed a manual segmentation assessment to assess their performance and calculate inter-rater and intra-rater reliability. The assessment was organized similarly to that of the training protocol, with tracers tracing on a set of seven subjects, waiting a week, then tracing on those same seven subjects. However, unlike the training protocol, the tracers did not receive feedback on their segmentations. The seven subjects chosen for this assessment were different from the subjects used in the training protocol and reflected a diverse set of lesion sizes and locations (Figure 3). The average lesion volume between subjects ranged from 1,174 to 32,419 voxels. During each tracing phase, tracers completed all seven subjects within 3 days. When they were fully finished with Phase 1, they took a 1 week break before beginning Phase 2. In Phase 2, tracers did not reference any of their segmentations drawn in Phase 1. After all 6 tracers completed Phase 2, their mask files were used to calculate inter-rater and intra-rater reliability.

### Inter-rater reliability measurement

Inter-rater reliability scores were calculated between all 6 tracers who completed the training. This measurement reflects the stability of the tracing protocol in developing standardized segmentations. Calculations were completed for the 7 T1w images used during the testing protocol for each phase on two different metrics: intraclass correlation coefficient (ICC) of the lesion volume and Dice similarity coefficient (DC). ICC was calculated using the R statistical package “psych” and we have reported ICC3k (in which a fixed set of k judges are assumed to rate each target), based on guidelines for interrater and intrarater reliability (Koo and Li, 2016). DC is an estimation of segmentation accuracy ranging from 0 to 1 that involves comparing the overlap between two masks. A result of 0 means there were no overlapping voxels, and a result of 1 means voxels overlapped identically. It differs from lesion volume, as two masks that have the same lesion volume could have a DC of 0 if they encompass completely different locations. A final averaged DC score was calculated through taking the average of each tracer's score compared to each other tracer, then permutated through every tracer, subject and phase. These averages were then averaged to create one final score.

### Intra-rater reliability measurement

Intra-rater reliability scores were calculated for all 6 tracers who completed the assessment and were averaged to produce a mean measure of intra-rater reliability. The intra-rater reliability score reflects the ability of the tracers to produce consistent masks over time. Calculations were completed for all 7 subjects within the testing protocol on the same two metrics: ICC and DC.

## Results

### Inter-rater reliability

Inter-rater reliability calculations demonstrated good reliability and confidence in the reproducibility of the protocol to create consistent results between different tracers. As shown in Table 1, ICC calculated from both phases was 0.98 (from lesion volume), indicating excellent inter-rater reliability based on published guidelines (Koo and Li, 2016). The overall average DC score (between both phases) is comparable to other published protocols for manual segmentation (Berron et al., 2017; Hashempour et al., 2019) and indicates good performance of the protocol (DC = 0.727). The discrepancy between ICC and DC is primarily explained through differences in which areas were included in the mask, particularly around border areas and areas with PVS and WMH.

**Table 1:** Inter-rater reliability.

	**Phase 1**	**Phase 2**	**Phase 1 + 2**
ICC3k	0.98	0.99	0.98
DC	0.716	0.739	0.727

Summary table demonstrating inter-rater reliability from ICC of lesion volume and average Dice coefficient. Scores are shown from lesion masks on each phase individually and combined.

### Intra-rater reliability result

ICC and DC were also calculated between Phase 1 and Phase 2 tracing in the testing assessment for each of the 6 tracers. Average values between all tracers are shown in Table 2 and demonstrate high intra-rater reliability of the training protocol (ICC = 0.99, DC = 0.839).

**Table 2:** Intra-rater reliability.

	**Rater 1**	**Rater 2**	**Rater 3**	**Rater 4**	**Rater 5**	**Rater 6**	**Average**
ICC3k	1	1	1.00	0.92	1	1	0.99
DC	0.857	0.811	0.865	0.809	0.857	0.837	0.839

Measure of intra-rater reliability, using comparison of ICC (based on lesion volume) for each rater and DC of each rater. Average ICC and DC are also noted.

### Visual quality control of segmentations

For each subject in the training protocol, segmentations were viewed for every tracer. Masks were layered on top of one another with lowered opacity on the T1w MRI to see discrepancies between each mask (Figure 3, right column).

## Discussion

Here, we share a protocol for manual lesion segmentation with the hope that it increases the reliability and reproducibility of segmentation across research groups. Although we acknowledge that manual lesion tracing is a subjective process, due to uncertainty in the images leading to difficult decision making (as well as human error), we anticipate that a written, detailed protocol can help reduce opportunities for error in future work. In addition, we emphasize that additional visual quality control, alongside use of this protocol, conducted by one or two outside raters, can strengthen the consistency between segmentations and reduce variance.

Using a standardized protocol can help to ensure consistency in manual segmentation processes across multiple researchers or research groups. Consistency is especially important when combining data from different locations in multi-site analyses, such as within the ENIGMA Stroke Recovery Working Group, which has currently harmonized data between 47 different research cohorts and completed manual lesion segmentation on over 1,300 high-resolution T1w MRIs. We anticipate that this standardized lesion tracing protocol may help research groups save time by not having to develop their own lesion tracing protocol and improve the possibility for more consistent lesion tracing across researchers.

We also hope that this protocol can be used as an educational resource for new researchers starting out in stroke research who need to perform manual lesion segmentation. In addition, we acknowledge that there may be many other protocols for manual segmentation. We hope that by providing this protocol, we can open a dialogue with other researchers about their own individual techniques for manual segmentation and promote reproducibility by sharing detailed methods. Detailed methods and published protocols can also help improve reproducibility and reliability across research sites. Within an increasing number of multi-site neuroimaging studies, research teams will rely on protocol resources to maintain the consistency needed for their data, especially within stroke imaging research. Protocols also facilitate public releases of multi-site large stroke neuroimaging datasets, such as ATLAS v1.0 (Liew et al., 2018) and v2.0 (Liew et al., 2022a).

## Author contributions

BT designed and conceptualized the study, role in the acquisition of data, analyzed and interpreted the data, drafted, and revised the manuscript for intellectual content. MD contributed to the design of the study, role in the acquisition of data, and revised the manuscript for intellectual content. GB contributed to the design of the study and revised the manuscript for intellectual content. S-LL designed and conceptualized the study, analyzed and interpreted the data, drafted, and revised the manuscript for intellectual content. All authors contributed to the article and approved the submitted version.
